# A Two-Step Single Plex PCR Method for Evaluating Key Colonic Microbiota Markers in Young Mexicans with Autism Spectrum Disorders: Protocol and Pilot Epidemiological Application

**DOI:** 10.3390/diagnostics13142387

**Published:** 2023-07-17

**Authors:** Julián Herrera-Mejía, Rocío Campos-Vega, Abraham Wall-Medrano, Florinda Jiménez-Vega

**Affiliations:** 1 Instituto de Ciencias Biomédicas, Universidad Autónoma de Ciudad Juárez, Anillo Envolvente del PRONAF y Estocolmo s/n, Ciudad Juárez 32310, Chihuahua, Mexico; al194425@alumnos.uacj.mx; 2 Programa de Posgrado en Alimentos del Centro de la República (PROPAC), Research and Graduate Studies in Food Science, School of Chemistry, Universidad Autónoma de Querétaro, Santiago de Querétaro 76010, Querétaro, Mexico; chio_cve@yahoo.com.mx

**Keywords:** *Faecalibacterium prausnitzii*, *Bacteroides fragilis*, *Desulfovibrio vulgaris*, *Akkermansia muciniphila*, microbiota, two-step PCR, 16S rRNA, autism, ASD

## Abstract

Many neurological disorders have a distinctive colonic microbiome (CM) signature. Particularly, children with autism spectrum disorders (ASD) exhibit a very dissimilar CM when compared to neurotypical (NT) ones, mostly at the species level. Thus far, knowledge on this matter comes from high-throughput (yet very expensive and time-consuming) analytical platforms, such as massive high-throughput sequencing of bacterial 16S rRNA. Here, pure (260/280 nm, ~1.85) stool DNA samples (200 ng.µL^−1^) from 48 participants [39 ASD, 9 NT; 3–13 y] were used to amplify four candidate differential CM markers [*Bacteroides fragilis* (BF), *Faecalibacterium prausnitzii* (FP), *Desulfovibrio vulgaris* (DV), *Akkermansia muciniphila* (AM)], using micro-organism-specific oligonucleotide primers [265 bp (BF), 198 bp (FP), 196 bp (DV), 327 bp (AM)] and a standardized two-step [low (step 1: °*Tm*—5 °C) to high (stage 2: °*Tm*—0 °C) astringent annealing] PCR protocol (2S-PCR). The method was sensitive enough to differentiate all CM biomarkers in the studied stool donors [↑ abundance: NT (BF, FP, AM), ASD (DV)], and phylogenetic analysis confirmed the primers’ specificity.

## 1. Introduction

Next-generation sequencing (NGS) has revolutionized the fields of personalized nutrition and precision medicine. Particularly, high-throughput sequencing of bacterial 16S ribosomal RNA (16S rRNA) is considered the “gold standard” method for profiling the colonic microbiota (CM) from stool samples [1]. The “CM signature” of many chronic diseases such as irritable bowel syndrome [2], type-1 diabetes [3], colorectal cancer [4], and CM deviations in pediatric patients with autism spectrum disorders (ASD) as compared to neurotypical (NT) ones [5,6,7,8,9], have been unveiled by NGS methods, confirming their superiority over conventional microbiological methods and traditional 16S rRNA gene polymerase chain reaction (PCR)-Sanger sequencing methods [1]. However, implementing NGS methods in clinical settings is time-consuming and expensive. Moreover, in certain cases, NGS requires certain set-up analysis/interpretation conditions that often result in the nonidentification of bacterial species with low fecal abundance [10,11].

The pediatric colonic microbiota (CM) comprises five main phyla (*Bacteroidetes*, *Firmicutes*, *Proteobacteria*, *Verrumicrobia*, and *Actinobacteria*) [12], and children’s neurodevelopment is accompanied by CM changes over time [13]. This apparently reduced diversity in microbial phyla contrasts with a high dynamism at the species level, in such a way that subtle bacterial deviations (dysbiosis) can mean an early sign of disease since certain bacterial members (commensals and/or pathogens) switch CM from healthy to nonhealthy [14]. ASD children also exhibit a CM enriched in these phyla [15,16,17,18], although qualitative (diversity) and quantitative (abundance) microbial alterations synergize with ASD pathophysiology [5,19]. *Faecalibacterium prausnitzii* (FP; *Firmicutes*), an anaerobic butyrate-producing bacteria, plays a pivotal role in gut-brain axis homeostasis, and its change rate is a common feature of many inflammation-related diseases [20,21] and behavioral problems [13]. *Bacteroides fragilis* (BF; *Bacteroidetes*) is an obligate anaerobic gram-negative bacillus that represents 1–2% of gut resident bacteria and poses immunostimulatory activity [22]. *Desulfovibrio vulgaris* (DV; *Proteobacteria*), a sulfate-reducing gram-negative bacteria [23], and *Akkermansia muciniphila* (AM; *Verrumicrobia*), a mucolytic gram-negative bacterium with probiotic and antiobesity effects [13,24,25]. These CM commensals have been proposed as CM differential markers in children with ASD vs. NT [5,6,7,8,9,17,18,19] or with other psychiatric disorders [26].

This brief report describes a rapid/reliable method to amplify four CM markers (BF, FP, DV, and AM) in stool samples from ASD and NT pediatric donors. It consists of a two-step single-plex polymerase chain reaction (PCR) and uses species-specific oligonucleotide primers targeting small 16S rRNA (v3-v4 region) sequences. The method could be an alternative to conventional microbiological and complex NGS-based methods. The pilot epidemiological application described herein, also adds new evidence to the scientific discussion on the clinical relevance of choosing species-specific markers of the CM in ASD vs. NT children [27].

## 2. Materials and Methods

### 2.1. Subjects

Pediatric patients previously diagnosed with ASD by experienced pediatric psychologists and neurologists, using the *Diagnostic and Statistical Manual of Mental Disorders* (DSM-V, 5th Ed.), were included in the ASD group (*n* = 39), while aged (3–13 y)/sex (male)-matched healthy subjects were included in the neurotypical (NT) group (*n* = 9). Candidates with other neurological, metabolic, genetic pathologies, or altered physiological conditions that may impact the subject’s CM were excluded. ASD participants were recruited from special education institutions and nongovernmental organizations serving children/youth with ASD and other neurodevelopmental disorders, in Ciudad Juarez, Chihuahua, Mexico (31°44′42″ N, 106°29′06″ W).

### 2.2. Ethics

This study was reviewed and approved by the Bioethics Committee of the Autonomous University of Ciudad Juarez (Authorization CIEB-2020-1-20) and was conducted in accordance with the Declaration of Helsinki (DoH; https://www.wma.net/what-we-do/medical-ethics/declaration-of-helsinki/ (accessed on 1 May 2020)) and Mexican regulations for clinical studies and biological waste handling and disposal [28]. Parents or legal guardians were aware of the whole study design, and written informed consent was obtained from them before participation.

### 2.3. Biological Samples

Fresh stool specimens (5–10 g) were collected into sterile containers by a participant’s relative after being given a detailed explanation of how to perform this procedure. Stool consistency was recorded with the Bristol scale, as suggested by [29]. Stool samples were kept at 4 °C during transport, delivered to the laboratory within 4 h, and aliquots (200 mg) from each specimen were individually frozen at −80 °C until use [29]. On the day of DNA extraction, stool samples (2.5 g) were thawed and homogenized in a saline solution (1:2 *w*/*v*, 0.9% NaCl) in falcon tubes, vortexed, and centrifuged (Eppendorf centrifuge 5804R 15-amp version, Eppendorf AG, Hamburg, Germany) at 2000 rpm, 5 min, 4 °C. Supernatants (~1.5 mL) were further transferred to microtubes (2 mL), centrifuged (Hermle Labnet Z216MK, Mandel Guelph, ON, Canada) at 13,000 rpm and 4 °C for five min, and the supernatant was discarded. The stool pellet was rinsed in saline (1 mL), vigorously homogenized, and centrifuged (13,500 rpm, 5 min, 4 °C), and this process was repeated twice. Recovered pellets were immediately used for DNA extraction.

### 2.4. DNA Extraction and Purification

It was performed by the conventional phenol: chloroform: isoamyl alcohol (PCI: 25:24:1 *v*/*v*) method [30]. Briefly, stool pellets were dissolved in lysis buffer (0.5 mL; 0.2 M Tris-HCl pH 8.0, 10 Mm EDTA, 0.5 M NaCl, and 1% SDS) with 5 µL of a proteinase K solution (50 mg.mL−1), vigorously shaken (twice), and incubated [round 1 (56 °C, 30 min), round 2 (100 °C, 10 min)] in a digital block heater and cooled afterward. Then, 400 µL of PCA solution was then added to each sample, vigorously shaken, and centrifuged (12,000 rpm, 3 min). The supernatant (DNA-containing fraction) was washed three times by adding 400 µL of phenol-chloroform solution (24:1), and DNA was further precipitated with ethanol (100%): sodium acetate solution (10:1 *w*/*v*, 400 µL), cooled (−20 °C) overnight, centrifuged (14,000 rpm, 15 min, 4 °C), washed in cold ethanol (75%), and centrifuged (7500 rpm, 5 min) once again. The DNA pellet was resuspended (30 µL of nuclease-free water), concentration and purity (260/280 nm, ~1.85) were measured spectrophotometrically (NanoDrop 2000; Thermo Fisher Scientific, Waltham, MA, USA), and standardized DNA samples (100 ng.µL^−1^) were stored at −80 °C until analysis.

### 2.5. Selection of CM Markers

All CM’s differential markers (at the species level; ASD vs. NT children/adolescents) were selected through a stepwise bibliographic search strategy using medical subject headings (MeSH), unique IDs, and Boolean operators. Briefly, recent systematic reviews and meta-analyses [6,7,8,9] on the studied subject [gastrointestinal (GI) microbiome and ASD] were reviewed to select four discriminant (ASD vs. NT) commensal bacterial species (BF, FP, AM, and DV). Afterward, a systematic search was carried out to identify the frequency of mentions of these CM markers in scientific articles published between January 1980 and May 2023. The three-stage search strategy (and subsequent bibliometric analysis) was performed as follows: (1) “Autism/autistic disorder (D001321)” OR “ASD (D000067877)”, (2) “GI microbiome (D000069196)” or “Microbiota (D064307)”, (3) and one of the following bacteria: “*Bacteroides fragilis*” (BF; D001441), “*Akkermansia muciniphila*” (AM; C000647304), “*Faecalibacterium prausnitzii*” (FP; D000070037) or “*Desulfovibrio vulgaris*“(DV; D016969). Two authors (JHM/AWM) extracted and analyzed data from Google Scholar (https://scholar.google.com/ (1 January 2023)), differences were fully discussed, and any conflict was resolved by a third author (F.J.V.). Lastly, a manual selection of key studies using “high throughput nucleotide sequencing (D059014)” or “gene sequencing” and “16S rRNA (D012336)” MeSH terms was initially screened to select those reporting at least one of the above commensal bacteria and was used to systematically document intergroup (ASD vs. NT) differences.

### 2.6. Oligonucleotide Primers of 16S rRNA

Custom-made primers (Integrated DNA Technologies, Coralville, IA, USA) targeting 16S rRNA gene (v3–v4 regions) fragments of all four CM markers [*Bacteroides fragilis* (BF; 265 bp), *Faecalibacterium prausnitzii* (FP, 198 bp), *Desulfovibrio vulgaris* (DV, 196 bp), and *Akkermansia muciniphila* (AM, 327 bp)] are listed in Table 1. Primers used to detect FP and AM were selected from the literature [19,20,25], while those for BF and DV were in-house designed with the PRIMER-Blast software (https://www.ncbi.nlm.nih.gov/tools/primer-blast/ (accessed on 1 December 2022)) using NCBI-deposited sequences [BF (NR_074784.2), DV (AB252583.1)] and performed at default settings [31]. The specificity of all primers was checked against NCBI-deposited sequences for the same bacteria using the *Basic Local Alignment Search* (BLAST) software. Phylograms (sequence homologies with four closest sequences) were constructed using the Fitch–Margoliash (FM) and neighbor-joining algorithms, using the Clustal-W tool of the BioEdit sequence alignment editor v. 7.2 (https://bioedit.software.informer.com/7.2/ (accessed on 1 December 2022)), using default parameters.

### 2.7. Two-Step Single-Plex PCR (2S-PCR)

**Figure 1:**
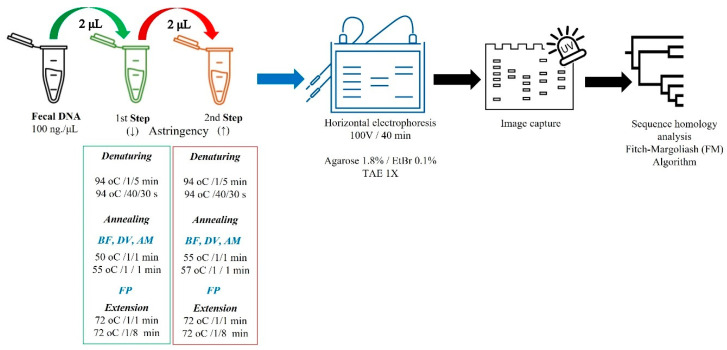
Two-step single-plex PCR (2S-PCR) protocol. *Akkermansia muciniphila* (AM), *Bacteroides fragilis* (BF), ethidium bromide (ETBr), *Desulfovibrio vulgaris* (DV), and *Faecalibacterium prausnitzii* (FP).

The single-plex 2S-PCR protocol used here consisted of two consecutive runs differing in annealing melting temperatures yet using the same primer set (Figure 1). 

Briefly, *Stage 1* [forty cycles; ↓ astringency (°*Tm* −5 °C)] and *Stage 2* [forty cycles; ↑ astringency (at °*Tm*)]. *Stage 1*: The reaction mixture (20 µL) consisted of a 2-µL (200 ng) DNA template, 12 µL of GoTaq^®^ (DNA polymerase, dNTPs, MgCl_2_, and reaction buffer), sense (Fw)/antisense (Rv) oligonucleotide primers (1 µL, 200 µM each), 4 µL PCR-grade water. Polymerization reactions were performed in a ProFlex^TM^ PCR system (Applied Biosystems, Forest City, CA, USA), and thermocycling conditions (denaturing/annealing/elongation) are described in Figure 1. *Stage 2*: 2 µL of 16S rRNA-amplified product was mixed with the same reaction mixture (total volume 20 µL) and amplified with a higher astringency (annealing temperature; Figure 1). Afterward, the molecular weight marker (DNA ladder 100-bp, Promega Cat# G2101) and PCR amplicons (3 µL) were electrophoretically analyzed on agarose gels (1.8%) supplemented with ethidium bromide (EtBr, 0.1%), using 1x TAE Buffer (40 mM Tris-acetate, 1.0 mM Na_2_EDTA, pH 8.3) as the carrier. Electrophoresis was performed for 40 min at 100 V, gels were viewed using a UV transillumination system, the images were digitized (1.5–2.5 exposure) using a Kodak EDAS 290 system, and the densitometric analysis was performed with the Kodak 1D Image software Ver. 3.6 (Kodak, Rochester, NY, USA). PCR amplification products were stored at −20 °C until use if the electrophoretic run was not performed immediately. Lastly, DNA titration curves using samples with confirmed high and low bacterial (AM, BF, DV, FP) abundance (16S rRNA gene) were titrated at ranged concentrations (0, 12.5, 25, 50, 100, and 200 ng.µL^−1^) to confirm the accuracy of using 200 ng.µL^−1^ as the preset DNA template (Figure 1).

### 2.8. Statistics

Intergroup comparisons (ASD vs. NT) of the relative fecal abundance (as optical density/arbitrary units, OD/AU) of each bacterial species (BF, FP, DV, and AM) were performed with the nonparametric Mann–Whitney U test. *p* < 0.05. Graphs and statistical analysis were performed with the GraphPad Prism 8.0.2 software (Graph pad software, San Diego California, USA).

## 3. Results

### 3.1. Selection of CM Differential Markers

All four commensal bacteria studied here were considered differential CM markers between NT and ASD children. The rationale came from an initial bibliometric analysis (Table 2) indicating that: (A) The number of publications related to “GI microbiome (MeSH unique ID: D000069196)” or “Microbiota (D064307)” in people with “Autism/autistic disorder (D001321)” or “ASD (D000067877)” has grown significantly in the past two decades (Table 2), and (B) a concurrent growth in research/review articles reporting all four CM markers under study also increased (X^2^-Polynomial) from 2010 onwards (BF > FB > AM > DV). 

Such growth in the number of mentions was closely followed by evidence-based driven data from high-throughput nucleotide sequencing (D059014) of the prokaryote 16S rRNA gene (D012336), showing that the relative abundance of these four bacterial species was indeed differential (either ↑ or ↓) in the CM from NT and ASD children (Table S1), although reports on similar abundance have also been reported to a lesser extent (Table S2).

### 3.2. Primer Design and Analysis

The 16S rRNA oligonucleotide primers used in this study (Table 1) were either reported previously by others (FB, AM) or in-house designed from Genebank-deposited sequences (BF, DV). All primers’ Tm (°C) and GC content (%) ranged from 55 to 60 and 50 to 65, respectively. The BLAST analysis of each amplicon [BF (265 bp), FP (198 bp), DV (196 bp), AM (327)] indicated ≥90% identity with each corresponding target gene (16S rRNA), and FM-phylograms (Figure S1) confirmed that all amplicon sequences were close [distance difference (ADD, %): 0.2 (FP) to 5.1 (AM), sum of squares (ΣSQ): 0.000 (FP, BF)—0.495 (AM)] to four Genebank-deposited sequences for the same bacterial strain.

### 3.3. 2S-PCR

**Figure 2:**
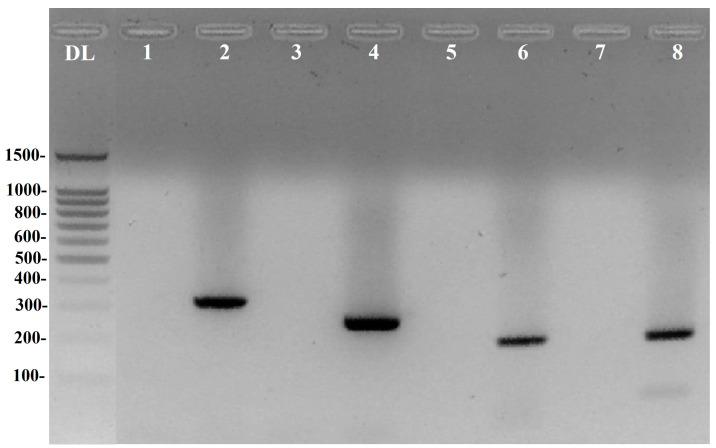
Agarose (1.8%) gel electrophoresis of two-step PCR DNA amplicons. DNA ladder (DL; 100-bp, Promega Cat# G2101), *Akkermansia muciniphila* (AM*,* 327 bp; Lanes 1–2), *Bacteroides fragilis* (BF*;* 265 bp; Lanes 3–4), *Faecalibacterium prausnitzii* (FP*;* 198 bp; Lanes 5–6), *Desulfovibrio vulgaris* (DV, 196 bp; Lanes 7–8) 16s rRNA amplicons coming from the two-step PCR protocol [Low (odd lanes)/high (even lanes) astringent conditions (↓ vs. ↑ °*Tm*)]. See Figure 1 and text for details.

The two-step PCR protocol used in this study (Figure 1) led to the selective amplification of all target genes (Figure 2). As shown in Figure 2, amplicons were detected at enough concentration (OD/AU) in the 2nd stage [↑ annealing astringency, Lanes 2 (AM), 4 (BF), 6 (FP), and 8 (DV)] from a ↓ astringent pre-amplified sample [Lanes 1 (AM), 3 (BF), 5 (FP), and 7 (DV)] and 100–200 ng.µL^−1^ of DNA template is enough to ensure the amplification of all four (AM, BF, DV, and FP) targeted 16s rRNA genes at detectable levels (Figure S2).

### 3.4. CM Commensal Biomarkers in ASD and NT Pediatric Patients

**Figure 3:**
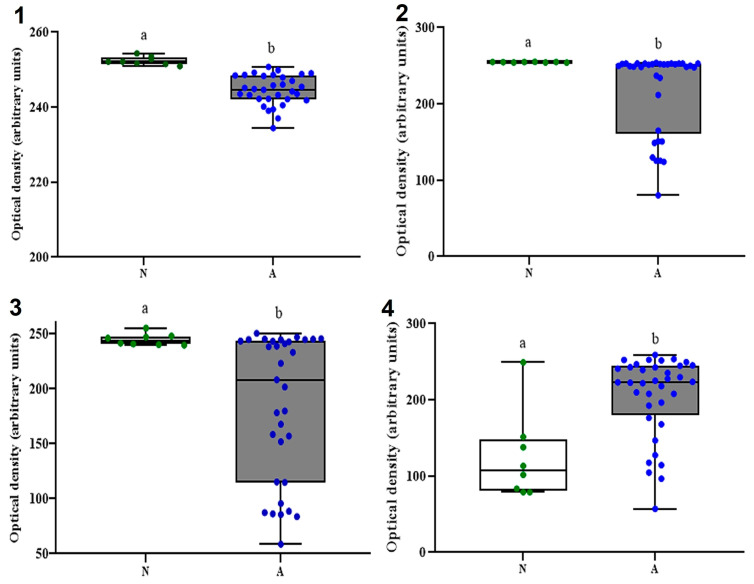
Gene quantification of 16S rRNA targeting four colonic microbiota members in stools from young Mexicans with autism spectrum disorders and neurotypical. *F. prausnitzii* (**1**), *B. fragilis* (**2**), *A. muciniphila* (**3**), and *D. vulgaris* (**4**). Each box plot [neurotypical (N; *n* = 9), autism spectrum disorders (A; *n* = 39) patients] extends from the 25th–75th percentile (whiskers), median value (inner horizontal line), and individual values (green/blue dots) showing the highest and lowest values, excluding outliers. Different superscript letters indicate statistical differences between N and A groups (Mann-Whitney U test, *p* < 0.05).

Differences in the relative abundance (as OD/AU) between the ASD (*n* = 39) and NT (*n* = 39) groups for all four commensals are depicted in Figure 3 (group data) and Figure S3 (individual data). As expected, all four studied CM performed differentially (↑ abundance) between N (FP, BF, and AM) and ASD (DV). Particularly, while all NT participants showed mean OD/AU <1999 for DV (including two undetected), 66% of all ASD participants showed values >199, with just one participant (2.6%) undetected (Figure S3).

## 4. Discussion

The increasing recognition that CM plays a relevant role in the natural history (genesis-progression-resolution) of many GI diseases (e.g., irritable bowel syndrome [2] and colorectal cancer [4]) or neurological/psychiatric disorders, such as ASD [5,6,7,8,9,17,18,19] and others [26], has prompted basic research and the development of NGS in the last two decades [32]. High-throughput massive gene sequencing platforms (e.g., Illumina Hi/MiSeq) have increasingly been used to investigate gene expression patterns (endpoint or progressive) of key molecular markers in health vs. disease conditions, either targeting hosts’ [33] or the microbiome’s [34] genomic traits. Particularly, the NGS of the prokaryotic 16S rRNA gene, a reliable marker for more than 97% of bacterial species [35], reduces the analytical shortcomings of culturing strict/facultative anaerobic bacteria by microbiological methods or traditional 16S rRNA PCR-Sanger sequencing methods [1,36]. 

The relative abundance of 16S rRNA PCR products (amplicons) generated by NGS is later translated into microbiome composition (at all taxonomic levels) by means of bioinformatic tools [33,34]. However, implementing NGS methods in clinical settings is not only expensive but complicated and often results in the nonidentification of scarcely abundant bacterial species in CM [10]. Moreover, in deciphering CM’s taxonomic complexity, NGS techniques are sometimes combined with conventional two-step PCR amplification methods [11,30], such as that reported here. Oligonucleotide primers targeting the 16S rRNA gene (V3-V4 region) for the four CM commensals studied here, proved to be sensitive/specific enough for such a purpose (Figure 2 and Figure S1). From a molecular standpoint, the 16S rRNA gene possesses very conserved kingdom-specific regions (commonly used to design “universal primers”) and nine hypervariable regions (V1–V9) randomly used for identifying operational taxonomic units (OTUs) in CM’s phylogenetic studies [33,34,35,36]. Restricting amplification of the V3–V4 regions of this gene helps to differentiate CM’s bacterial taxa, although choosing primers targeting other regions may lead to different analytical results [20,36,37]. 

The primer set used to identify FP was designed by Wang et al. [38] to identify a fragment (199 bp) of the 16S rRNA gene of *Fusobacterium prausnitzii* (ATCC 27766/27768) in both human and rat stool samples with very high PCR titers, a fact that later helped to conclude that this commensal is the second largest colonizer in the human gut [20]. The same primer set was later used for the differential identification (abundance) of this bacterium in samples from children with ASD and NT [19]. It is worth mentioning that Tanno et al. [20] recently showed that this primer set allows the amplification of all FP genogroups when compared to other primer sets previously reported. The primer set used to identify AM was initially reported by Derrien et al. [39], and recently used to map differences in stool samples from ASD (↓) vs. NT (↑) children [25] and Mexican children with (↓) and without (↑) metabolic disorders [40]. Primer sets used to detect BF and DV were in-house designed (Table 1), and their size (18–20 nucleotides), % GC (50–58%), and *Tm* (~55 °C) were within recommended ranges [31,41]. The primer set used to amplify the 196-bp fragment of DV’s 16S rRNA, even though its specificity was bioinformatically demonstrated (Figure S1), also caused the simultaneous amplification of a shorter fragment (<100 bp). When trying to amplify short DNA fragments (of any origin) from complex samples (e.g., feces), additional PCR fragments (usually smaller in size) may occur due to multiple factors, including the number of amplification cycles and optimal annealing temperature [41]. It has also been seen that additional steps for rRNA enrichment (as occurred in the second-stage PCR) may lead to PCR-sequence artifacts, introducing semi/quantification errors and difficulties in taxonomic profiling [42]. Additionally, DV’s 16S rRNA genogroups are as diverse as those from FP [20], in such a way that intra- and intersubject variability (presence and relative density) are high [43], a fact that further complicates its semi/quantitative analysis. Whether these arguments indeed justify our results or if both (BF’s or DV’s) designed primer sets perform the same as others previously reported deserves further study.

The single-plex 2S-PCR protocol reported here just differed in annealing temperatures [Stage 1, (°*Tm* −5 °C); Stage 2 (°*Tm*)], resulting in target-specific/amplicon-rich samples with enough intergroup (ASD vs. NT) differential resolution; such fine-tuning from low-(stage 1)-to-high (stage 2) astringent annealing temperatures resulted in enough amplicon amounts detectable by conventional ETBr stained-agarose gel electrophoresis [11,30]. Two-step PCR methods have proven to be a reliable way of tracking changes or differences in 16S rRNA gene phylotype abundances [11,44] and selecting an optimal annealing temperature between consecutive PCR runs, which improves the specificity and enrichment level of PCR products [42]. An optimal annealing temperature should be low enough to allow both primers (sense/antisense; F/R) to bind to the template without leading to the formation of nonspecific duplexes or intramolecular hairpins [41] and GC-rich rather than AT-rich primers will require a higher annealing temperature, although other PCR reagents and parameters could modify the selected °*Tm* [45]. 

It is noteworthy that a large amount of pure (260/280 nm, ~1.85) DNA template was used in the first PCR stage to increase the odds of a successful (detectable) amplification in the second stage. DNA titration curves confirmed that 100–200 ng.µL^−1^ of DNA template was sufficient to ensure the amplification of all four CM differential markers (AM, BF, DV, and FP) in a bacteria-specific manner. It is customary to use 10–50 ng of DNA template when: (A) amplifying multicopy target genes from noncomplex samples (e.g., bacterial cultures), (B) employing high-throughput output analytical platforms (e.g., real-time PCR, NGS), and (C) using amplifying signal probes (e.g., fluorescent probes). Conversely, when trying to amplify (16s rRNA) microbial species of low abundance (e.g., parasites, rare gut commensals) from molecularly complex samples (e.g., tissue biopsies, stool samples), from organic samples that may contain PCR inhibitors, or when using conventional PCR methods as those used here, a much higher amount (up to 20 times more) is often needed [42,46,47]. Data from DNA titration curves (Figure S2) seem to indicate that 100 ng.µL^−1^ could be enough to achieve a successful identification of all but BF in stool samples with log-low bacterial abundance (linear behavior up to 200 ng.µL^−1^), as compared to those with high bacterial abundance (X^2^-like behavior, plateau from 100 to 200 ng.µL^−1^ of DNA template). 

The simple and easy-to-implement 2S-PCR reported here was sensitive enough to differentiate all CM biomarkers in the studied stool. Our pilot results indicate a higher relative abundance of BF, FP, and AM in NT subjects, while the opposite occurred for DV (Figure 3). In several systematic reviews and/or meta-analyses [5,6,7,8,9], it has been reported that the CM of pediatric patients with ASD is dissimilar to that of NT ones; in these reports, it has also been postulated that the bacterial commensals reported here are differential markers for the presence and chronicity of both ASD and gastrointestinal illnesses [5,6,7,8,9,17,18,19]. It is noteworthy that DV’s abundance is closely related to the presence of inflammatory bowel disease (IBD) in a genogroup-specific manner [43], but its differential abundance in ASD vs. NT children remains debatable (Tables S1 and S2), even though IBD is quite common in ASD. Lastly, it is noteworthy that practically all this accumulated knowledge comes from NGS analysis of bacterial 16S rRNA in stool samples from pediatric populations living in the United States, Asia, and Europe, yet scarcely from Latin American countries [48].

However, without making an in-depth discussion, it is worth noting that even today the controversy continues about whether these markers increase or decrease (Table S1) or remain similar (Table S2) between patients with and without ASD. For example, *Desulfovibrio* spp. and *Akkermansia* spp. have been reported to be either lower [25] or higher [49] in ASD children as compared to NT ones, and similar conflicting results have been reported for *Bacteroides* spp. and *Faecalibacterium* spp. [7,50,51]. Factors associated with such discrepancies are the age and number of participants, enrollment criteria, ASD’s chronicity, selection of PCR method (e.g., conventional, NGS), and dietary patterns of participants with and without ASD [25,38,45,50,52], to name a few. Nevertheless, the putative physiological role (offensive or protective) of these CM markers on the gut-brain axis and ASD symptomatology (worsening or ameliorating) has been perfectly stated [20,21,22,23,24,25], and so a robust conclusion on this matter is very close to being resolved.

As previously stated, NGS methods are the “gold standard” against which any in-house developed method, such as the one reported here, must be validated. Since the first report on the use of 16S rRNA gene profiling in bacterial phylogenetic analysis [53], the development of NGS platforms has grown exponentially, and some of them are currently cheaper than preceding ones [42], leaving aside any traditional amplification methods to detect this prokaryotic gene in complex samples. However, metagenomic sequencing of pediatric gut microbiomes using NGS platforms typically relies on reference databases, challenging the identification of specific microbes [54]. Moreover, implementing NGS methods in clinical settings is far from being extensively implemented, particularly in countries with moderate-to-poor resources for primary healthcare and clinical research. As for this study, even though there have been significant advances in characterizing CM in children/adolescents living with ASD (as compared to NT), the definition of its basic features and, above all, the evaluation of interventions aimed at modifying it require lower-cost analytical strategies with sufficient analytical sensitivity and discrimination power. Lastly, the authors also acknowledge that this study has limitations that need to be addressed in the near future: (A) sequencing of all amplified products, particularly those generated with in-house designed primers (DV, BF [22,23]), (B) to test the method’s sensitivity under optimizing conditions (DNA-template gradients vs. # PCR cycles), (C) to co-amplify conserved regions of the prokaryotic 16S rRNA using “universal primers” to switch from OD/AU to “normalized units” (semiquantification), (D) to migrate this “single plex” endpoint 2S-PCR protocol to a multiplex assay and to evaluate its sensitivity/specificity vs. RT-PCR, and (E) to evaluate the effect of nonanalytical factors associated with the response to the developed method [35,41].

## 5. Conclusions

In this work, a two-step PCR method was developed for the selective/sensitive amplification of four MC markers with differential expression between pediatric patients with and without autism. The method turned out to be sensitive and selective enough to complement more complex metagenomic sequencing studies of the 16S rRNA gene (NGS) or as a fast and low-cost alternative for the rapid assessment of bacterial species with bioactivity (deregulatory or positive) on the microbiota-brain axis, potentially managed with personalized nutrition protocols (neuronutrition).

## Figures and Tables

**Table 1:** Oligonucleotide primers of 16S rRNA.

Bacteria	Primer Sequences (3′-5′)	Length (bp)	*Tm* (°C)	GeneBank/Ref.
BF	*F*: CCCTTTACTCGGGGATAG *R*: CTTGGCTGGTTCAGGCTAG	265	55	NR_074784.2 *
FP	*F*: GATGGCCTCGCGTCCGATTAG *R*: CCGAAGACCTTCTTCCTCC	198	57	[19,20]
DV	*F*: GCGTGAAAGGACTTCGGT *R*: CCACCAACTAGCTAATGGGA	196	55	AB252583.1 *
AM	*F*: CAGCACGTGAAGGTGGGGAC *R*: CCTTGCGGTTGGCTTCAGAT	327	60	[25]

*Bacteroides fragilis* (BF), *Faecalibacterium prausnitzii* (FP), *Desulfovibrio vulgaris* (DV), *Akkermansia muciniphila* (AM), primer melting temperature (*Tm* °C). Forward (sense, F), reverse (antisense, R). GenBank (https://www.ncbi.nlm.nih.gov/genbank/ (accessed on 1 December 2022)) accession number (*).

**Table 2:** Bibliometric analysis 1980–2023 ^1^.

MeSH-Keyword, BOP, (Search Stage) ^2^	80–89	90–99	00–09	10–19	20–23
Autism/autistic disorder/ASD (1)	27,300	68,900	321,000	853,000	211,000
(1) *+* Microbiota/GI microbiome (2)	16	46	996	12,800	16,800
(1 + 2) + *Bacteroides fragilis* (3a)	4	1	38	1590	1670
(1 + 2) + *Akkermansia muciniphila* (3b)	0	1	8	922	1950
(1 + 2) + *Faecalibacterium prausnitzii* (3c)	0	0	17	1080	1670
(1 + 2) + *Desulfovibrio vulgaris* (3d)	0	0	6	105	117

^1^ Google Scholar (https://scholar.google.com/ (accessed on 1 December 2022)), filtered by 10-y ranges. ^2^ Boolean operators (*BOP*): OR (/) AND (+) Autism spectrum disorder (ASD), gastrointestinal (GI).

## Data Availability

Dataset used and/or analyzed during the correct study are included in the manuscript.

## Supplementary Materials

The following supporting information can be downloaded at: https://www.mdpi.com/article/10.3390/diagnostics13142387/s1. Table S1: Selected evidence on the relative comparison of four colonic microbiota members in neurotypical (NT) and autism spectrum disorder (ASD) pediatric patients from next-generation sequencing (NGS) methods: supporting evidence (*p* < 0.05), Table S2: Selected evidence on the relative comparison of four colonic microbiota members in neurotypical (NT) and autism spectrum disorder (ASD) pediatric patients from next-generation sequencing (NGS) methods: nonsupporting evidence (*p* > 0.05). Figure S1: Phylogenetic (sequence homology) analysis, sequence inter-relationship of *B. fragilis* (BF*), *F. prausnitzii* (FP*), *A. muciniphila* (AM*)*, and D. vulgaris* (DV*) 16S rRNA amplicon sequences against the nearest NCBI-deposited sequences (same bacteria) were calculated and compared using the Fitch–Margoliash (FM) and neighbor-joining algorithms using the Clustal-W tool of the BioEdit sequence alignment editor v. 7.2 (https://bioedit.software.informer.com/7.2/ (accessed on 1 December 2022)), using default parameters. Distance difference (ADD), sum of squares (ΣSQ); Figure S2: DNA titration curves; Figure S3: Detection of bacteria species in fecal samples of neurotypical and autistic children identification of bacteria by RT-PCR. Each column represents a patient. Negative (white boxes) and positive (gray boxes) samples. Color intensity represents relative abundance, according to mean optical density values. *F.p* = *Faecalibacterium prausnitzii; B.f = Bacteroides fragilis; A.m = Akkermansia muciniphila; D.v = Desulfovibrio vulgaris.*
