# Supplementary material for: A Two-Step Single Plex PCR Method for Evaluating Key Colonic Microbiota Markers in Young Mexicans with Autism Spectrum Disorders: Protocol and Pilot Epidemiological Application
Source: Diagnostics (Basel). 2023 Jul 17;13(14):2387. doi: 10.3390/diagnostics13142387 (PMC10377852; doi:10.3390/diagnostics13142387)
Supplement: Supplementary file 1 [file diagnostics-13-02387-s001.zip › diagnostics-2445870-supplementary.pdf]

Supplementary materials

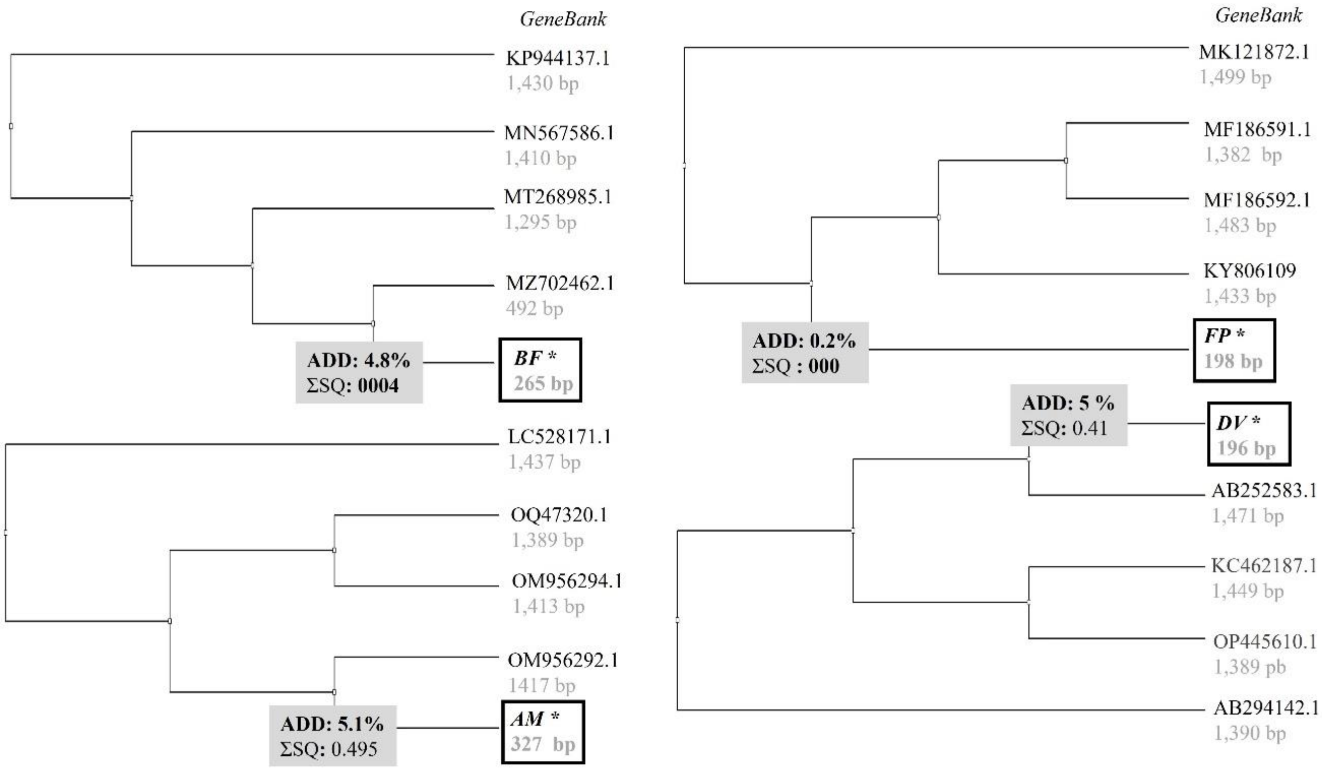

Figure S1. Phylogenetic (sequence homology) analysis

Sequence interrelationship of *B. fragilis* (BF*), *F. prausnitzii* (FP*), *A. muciniphila* (AM*), *D. vulgaris* (DV*) 16S rRNA amplicon sequences against the nearest NCBI-deposited sequences (same bacteria). Phylograms (sequence homologies) were constructed using the Fitch-Margoliash (FM) and Neighbor-Joining algorithms using the Clustal-W tool of the BioEdit sequence alignment editor v. 7.2 (https://bioedit.software.informer.com/7.2/), using default parameters. Distance difference (ADD), Sum of squares (ΣSQ).

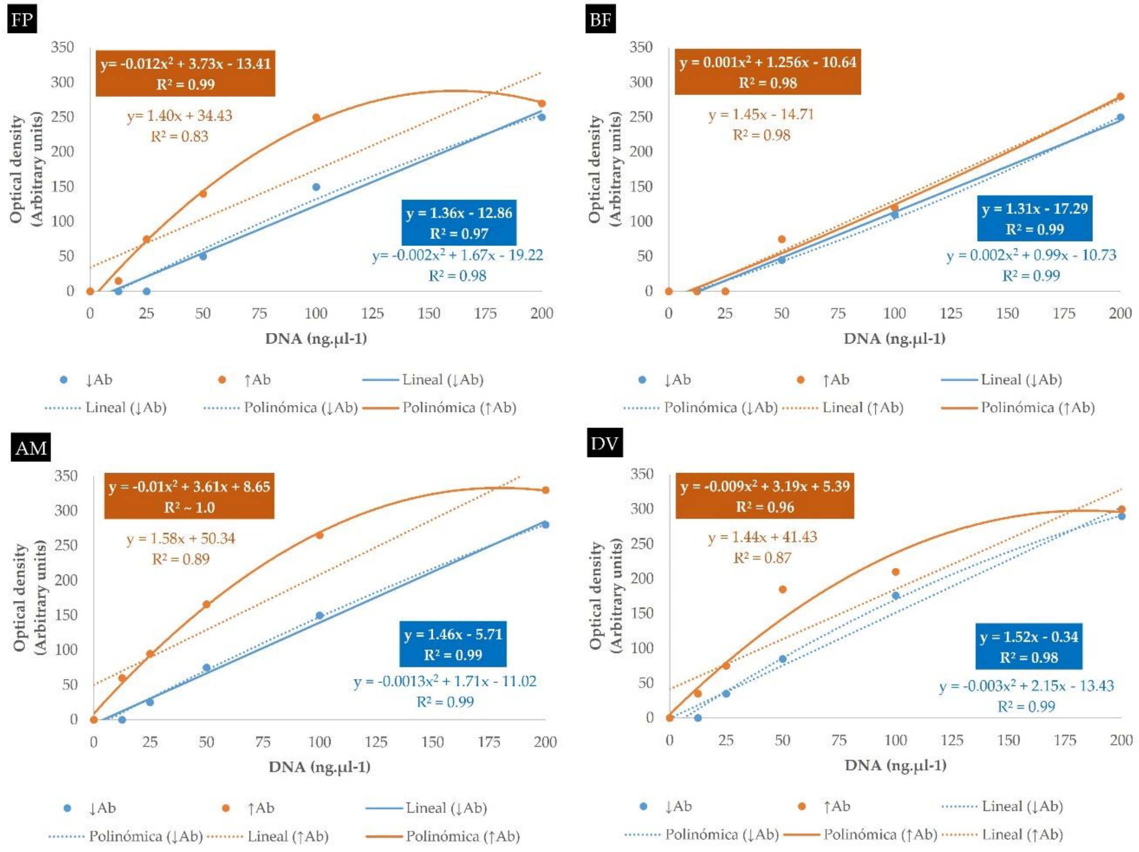

Figure S2. DNA titration curves. Total DNA extracted from samples with confirmed high (↑) and low (↓) bacterial [*Faecalibacterium prausnitzii* (FP), *Bacteroides fragilis* (BF), *Akkermansia muciniphila* (AM), *Desulfovibrio vulgaris* (DV)] relative abundance (pre-tested at 200 ng.µl^{-1} of DNA template), were titrated at ranged concentrations (0, 12.5, 25, 50, 100, and 200 ng.µL^{-1}). Test conditions in stages 1 and 2 are described within the text and **Figure 1**. Best goodness-of-fit (GOF) derived equation in brown (↑) and blue (↓) rectangles.

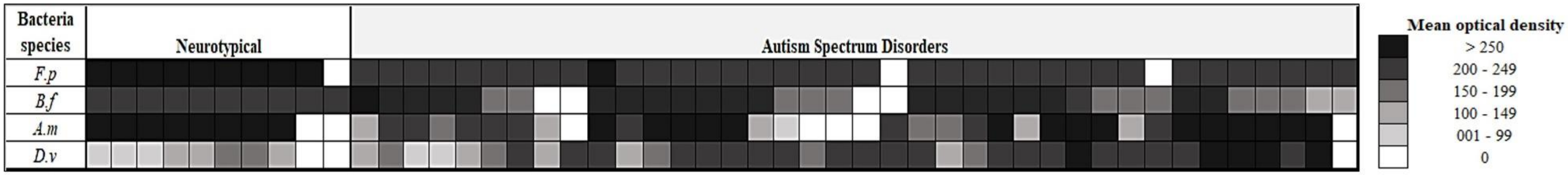

Figure S3. Detection of bacteria species in fecal samples of neurotypical and autistic children identification of bacteria by RT-PCR. Each column represents a patient. Negative (white boxes) and positive (gray boxes) samples. Color intensity represents relative abundance, according to mean optical density values. *F.p = Faecalibacterium prausnitzii; B.f = Bacteroides fragilis; A.m = Akkermansia muciniphila; D.v = Desulfovibrio vulgaris.*

Table S1. Selected evidence on the relative comparison of four colonic microbiota members in neurotypical (NT) and autism spectrum disorder (ASD) pediatric patients from next-generation sequencing (NGS) methods: : Supporting evidence ($p<0.05$)[1]

Country	NT/ASD	Experimental conditions [2]	BF	FP	DV	AM	Reference[5]
China	773/429	(--)/ Multiplex seq (v4) / Illumina platform	--	↓*	--	--	**[a]**
China	50/77	CTAB/qPCR (v4) / Illumina platform	--	↓*	--	--	**[b]**
USA	23/21	Power soil/qPCR (v2-v3)/Illumina platform	↑[3]	↓	--	↓	**[c]**
India	30/30	(--)/qPCR (v4)/ Illumina platform	↓[3]	↓	--	↓	**[d]**
China	6/35	(--)/Illumina platform	--	↓[4]	--	--	**[e]**
China	48/48	QiAMP/qPCR (v3-v4) / Illumina platform	↓	↓	--	↓	**[f]**
Italy	10/10	FastDNA® Pro-Soil/ bTEFAP (v1-v3)/ Illumina platform	↑	↓	--	↑	**[g]**
Italia	14/11	QiAMP/qPCR/ddPCR (v3-v4) / Illumina platform	↑[3]	↑*	--	--	**[h]**
China	18/71	SP Spin StooL/ NEXTFlex® (v1-v2) / Illumina platform	↓[3]	↑	--	↓	**[i]**
Japan	6/6	(--)/ qPCR/ddPCR (v3-v4) / Illumina platform	↑[3]	↑[4]	=	↑[4]	**[j]**
China	20/25	Omega soil/qPCR (v3-v4) / Illumina platform	↑[3]	↑[4]	--	--	**[k]**
China	60/138	QiAMP/qPCR (v3-v4) / Illumina platform	↑	↑*	↓[4]	--	**[l]**
Egypt	45/41	ISOLATE Fecal DNA/RT-PCR	↑[3]	--	=	--	**[m]**
Australia	22/23	bead-beating/ qPCR	↓*	↑	--	↓	**[n]**

[1] Increased (↑), same (=), decreased (↓), or not reported (--) abundance in ASD patients as compared to NT ones. [2] Nomenclature and experimental details detailed in the original study (see reference). [3] *Bacteroidaceae* (family). [4] At *genus* level. *Bacteroides fragilis* (BF), *Faecalibacterium prausnitzii* (FP), *Desulfovibrio vulgaris* (DV), *Akkermansia muciniphila* (AM). [5] Certain studies included in this table have been included at least once in four recent systematic reviews/metanalysis on the same topic: **[o-r]**. * Depending on the severity of gastrointestinal disorders.

Table S2. Selected evidence on the relative comparison of four colonic microbiota members in neurotypical (NT) and autism spectrum disorder (ASD) pediatric patients from next-generation sequencing (NGS) methods: Non-supporting evidence (p>0.05)[1]

Country	NT/ASD	Experimental conditions[2]	BF	FP	DV	AM	Ref
USA	6/29	MO BIO PS / NEXTFlex® (v1-v3-v4) / Illumina platform	↓[3]	=	=	=	**[s]**
China	143/143	(--)/ qPCR (v4) / Illumina platform	--	=	--	--	**[t]**
Korea	38/54	QiAMP/qPCR (v3-v4) / Illumina platform	↓[3]	--	--	--	**[u]**
India	24/30	QiAMP/qPCR (v3) / Illumina platform	↓[3]	↓[4]	↓[4]	=[4]	**[v]**
Russia	21/36	QiAMP/qPCR (v3-v4) / Illumina platform	=[3]	=[4]	--	=	**[w]**
China	45/45	QiAMP/qPCR (v3-v4) / Illumina platform	=[3]	=[4]	↓[4]	--	**[x]**
USA	44/59	QiAMP/qPCR (v1-v2/v1-v3) / Illumina platform	=[3]	=	--	--	**[y]**

[1]Increased (↑), same (=), decreased (↓), or not reported (--) abundance in ASD patients as compared to NT ones. [2] Nomenclature and experimental details detailed in the original study (see reference). [3]*Bacteroidaceae* (family). [4] At *genus* level. *Bacteroides fragilis* (BF), *Faecalibacterium prausnitzii* (FP), *Desulfovibrio vulgaris* (DV), *Akkermansia muciniphila* (AM). [5]Certain studies included in this table have been included at least once in four recent systematic reviews/metanalysis on the same topic: **[o-r]**. * Depending on the severity of gastrointestinal disorders.

References

a) Lou, M., Cao, A., Jin, C., Mi, K., Xiong, X., Zeng, Z., *et al.* Deviated and early unsustainable stunted development of gut microbiota in children with autism spectrum disorder. *Gut* **2022**, *71*(8), 1588–1599. https://doi.org/10.1136/gutjnl-2021-325115

b) Ding, X., Xu, Y., Zhang, X., Zhang, L., Duan, G., Song, C., *et al.* (2020). Gut microbiota changes in patients with autism spectrum disorders. *J. Psychiatric. Res.* **2020**, *129*, 149–159. https://doi.org/10.1016/j.jpsychires.2020.06.032

c) Kang, D.W., Ilhan, Z.E., Isern, N.G., Hoyt, D.W., Howsmon, D.P., Shaffer, M., *et al.* Differences in fecal microbial metabolites and microbiota of children with autism spectrum disorders. *Anaerobe* **2017**, *49*(2018), 121–131. https://doi.org/10.1016/j.anaerobe.2017.12.007

d) Agarwala, S., Naik, B., Ramachandra, N.B. Mucosa-associated specific bacterial species disrupt the intestinal epithelial barrier in the autism phenome. *Brain Behav. Immunity Health* **2021**, *15*(March), 100269. https://doi.org/10.1016/j.bbih.2021.100269

e) Zhang, M., Ma, W., Zhang, J., He, Y., Wang, J. Analysis of gut microbiota profiles and microbe-disease associations in children with autism spectrum disorders in China. *Sci. Rep.* **2018**, *8*(1), 13981. https://doi.org/10.1038/s41598-018-32219-2

f) Zou, R., Xu, F., Wang, Y., Duan, M., Guo, M., Zhang, Q., *et al.* Changes in the gut microbiota of children with autism spectrum disorder. *Autism Res.* **2020**, *13*(9), 1614–1625. https://doi.org/10.1002/aur.2358

g) De Angelis, M., Piccolo, M., Vannini, L., Siragusa, S., De Giacomo, A., Serrazzanetti, D. I., *et al.* Fecal microbiota and metabolome of children with autism and pervasive developmental disorder not otherwise specified. *PloS one* **2013**, *8*(10), e76993. https://doi.org/10.1371/journal.pone.0076993

h) Coretti, L., Paparo, L., Riccio, M. P., Amato, F., Cuomo, M., Natale, A., *et al.* Gut microbiota features in young children with autism spectrum disorders. *Front. Microbiol.* **2018**, *9*(Dec), 1–12. https://doi.org/10.3389/fmicb.2018.03146

i) Ye, F., Gao, X., Wang, Z., Cao, S., Liang, G., He, D., *et al.* Comparison of gut microbiota in autism spectrum disorders and neurotypical boys in China: A case-control study. *Synthetic Syst Biotechnol* **2021**, *6*(2), 120–126. https://doi.org/10.1016/j.synbio.2021.03.003

j) Inoue, R., Sakaue, Y., Sawai, C., Sawai, T., Ozeki, M., Romero-Pérez, G. A., *et al.* A preliminary investigation on the relationship between gut microbiota and gene expressions in peripheral mononuclear cells of infants with autism spectrum disorders. *Biosci. Biotechnol. Biochem.* **2016**, *80*(12), 2450–2458. https://doi.org/10.1080/09168451.2016.1222267

k) Ding, H., Yi, X., Zhang, X., Wang, H., Liu, H., Mou, W.W. Imbalance in the gut microbiota of children with autism spectrum disorders. *Front. Cell. Infection Microbiol.* **2021**, *11*(Nov), 1–9. https://doi.org/10.3389/fcimb.2021.572752

l) Chen, Z., Shi, K., Liu, X., Dai, Y., Liu, Y., Zhang, L., *et al*. Gut microbial profile is associated with the severity of social impairment and IQ performance in children with autism spectrum disorder. *Front. Psychiatry* **2021**, *12*, 789864. https://doi.org/10.3389/fpsyt.2021.789864

m) Ahmed, S. A., Elhefnawy, A. M., Azouz, H. G., Roshdy, Y. S., Ashry, M. H., Ibrahim, A. E., et al. (2020). Study of the gut microbiome profile in children with autism spectrum disorder: a single tertiary hospital experience. *J. Mol. Neurosci.* **2020**, *70*, 887-896. https://doi.org/10.1007/s12031-020-01500-3

n) Wang, L., Christophersen, C.T., Sorich, M.J., Gerber, J.P., Angley, M.T., Conlon, M.A. Low relative abundances of the mucolytic bacterium *Akkermansia muciniphila* and *Bifidobacterium spp.* in feces of children with autism. *Appl. Environ. Microbiol.* **2011**, *77*(18), 6718–6721. https://doi.org/10.1128/AEM.05212-11

o) Bezawada, N., Phang, T. H., Hold, G. L., Hansen, R. Autism spectrum disorder and the gut microbiota in children: a systematic review. *Ann. Nutr. Metab.* **2020**, *76*(1), 16-29. https://doi.org/10.1159/000505363

p) Liu, F., Li, J., Wu, F., Zheng, H., Peng, Q., Zhou, H. Altered composition and function of intestinal microbiota in autism spectrum disorders: a systematic review. *Trans. Psychiatry* **2019**, *9*(1), 43. https://doi.org/10.1038/s41398-019-0389-6

q) Iglesias-Vázquez, L., Van Ginkel Riba, G., Arija, V.,Canals, J. Composition of gut microbiota in children with autism spectrum disorder: a systematic review and meta-analysis. *Nutrients* **2020**, *12*(3), 792. https://doi.org/10.3390/nu12030792

r) Andreo-Martínez, P., Rubio-Aparicio, M., Sánchez-Meca, J., Veas, A., Martínez-González, A.E. A meta-analysis of gut microbiota in children with autism. *J. Autism Dev. Dis.* **2022**, *52*(3), 1374-1387. https://doi.org/10.1007/s10803-021-05002-y

s) Luna, R. A., Oezguen, N., Balderas, M., Venkatachalam, A., Runge, J. K., Versalovic, J., *et al*. Distinct microbiome-neuroimmune signatures correlate with functional abdominal pain in children with autism spectrum disorder. *Cmgh* **2017**, *3*(2), 218–230. https://doi.org/10.1016/j.jcmgh.2016.11.008

t) Dan, Z., Mao, X., Liu, Q., Guo, M., Zhuang, Y., Liu, Z., *et al*. Altered gut microbial profile is associated with abnormal metabolism activity of autism spectrum disorder. *Gut Microbes* **2020**, *11*(5), 1246–1267. https://doi.org/10.1080/19490976.2020.1747329

u) Ha, S., Oh, D., Lee, S., Park, J., Ahn, J., Choi, S., *et al.* Altered gut microbiota in korean children with autism spectrum disorders. *Nutrients* **2021**, *13*(10), 1–15. https://doi.org/10.3390/nu13103300

v) Pulikkan, J., Maji, A., Dhakan, D. B., Saxena, R., Mohan, B., Anto, M. M., *et al*. Gut microbial dysbiosis in Indian children with autism spectrum disorders. *Microb. Ecol.* **2018**, *76*(4), 1102–1114. https://doi.org/10.1007/s00248-018-1176-2

w) Averina, O.V., Kovtun, A.S., Polyakova, S.I., Savilova, A.M., Rebrikov, D.V., Danilenko, V.N. The bacterial neurometabolic signature of the gut microbiota of young children with autism spectrum disorders. *J. Med. Microbiol.* **2020**, *69*(4), 558–571. https://doi.org/10.1099/jmm.0.001178

x) Ma, B., Liang, J., Dai, M., Wang, J., Luo, J., Zhang, Z., *et al*. Altered gut microbiota in Chinese children with autism spectrum disorders. *Front. Cell. Inf. Microbiol.* **2019**, *9*, 40. https://doi.org/10.3389/fcimb.2019.00040

y) Son, J.S., Zheng, L.J., Rowehl, L.M., Tian, X., Zhang, Y., Zhu, W., *et al*. Comparison of fecal microbiota in children with autism spectrum disorders and neurotypical siblings in the simons simplex collection. *PloS one* **2015**, *10*(10), e0137725. https://doi.org/10.1371/journal.pone.0137725.
